# Acute toxic effects of *Bothrops atrox* venom on calcium homeostasis and bone tissue in mice

**DOI:** 10.1016/j.toxcx.2025.100237

**Published:** 2025-12-08

**Authors:** Hatem Kallel, Latifa Hamdaoui, Malek Elerou, Marwa Lakhrem, Stephanie Houcke, Majed Kammoun, Dabor Resiere, Tarek Rebai, Jean Marc Pujo, Ibtissem Ben Amara

**Affiliations:** a Intensive Care Unit, French Guiana University Hospital, Cayenne, French Guiana; b Tropical Biome and immunopathology CNRS UMR-9017, Inserm U 1019, Université de Guyane, French Guiana; c Laboratory of induced and developmental diseases LR19ES12, Sfax Faculty of Medicine, University of Sfax, 3029, Sfax, Tunisia; d Laboratory of Medicinal and Environment Chemistry, Higher Institute of Biotechnology, University of Sfax, PB 261, Sfax, 3000, Tunisia; e Intensive Care Unit, Martinique University Hospital, Fort de France, Martinique; f Emergency department, French Guiana University Hospital, Cayenne, French Guiana; g Amazin PopHealth, Département de Recherche et d’Innovation en Santé Publique (DRISP), Inserm Centre d’Investigation Clinique (CIC1424), French Guiana University Hospital, Cayenne, French Guiana

**Keywords:** *Bothrops atrox*, Venom, Bonne tissue, Bone remodeling, Mice

## Abstract

*Bothrops atrox* is a terrestrial pit viper inhabiting the Amazon region. The venom of *B. atrox* acts almost immediately at the site of the bite, leading to significant tissue damage, but also affects different organs. The present study investigated the acute impact of intraperitoneally administered *B. atrox* venom on bone tissue integrity and calcium homeostasis in mice. Plasma, bone homogenate, and urine samples from adult male and female Swiss mice (30 ± 2 g/mouse) were analyzed to assess calcium and phosphorus levels. Additionally, we examined bone oxidative stress parameters alongside histological and scanning electron microscopy (SEM) analysis. Our findings showed that *B. atrox* envenoming results in profound phosphocalcic homeostasis disturbances with hypercalcemia, hypophosphatemia, and decreased calcium and phosphorus bone content. We also observed increased reactive oxygen species and malondialdehyde, and consumption of antioxidants. Histological examination and SEM of bone tissue revealed thinning and discontinuity of trabecular bone and a significant reduction in intertrabecular links. In conclusion, *B. atrox* envenoming profoundly impacts bone metabolism and structural integrity in mice. The venom induces substantial alterations in calcium and phosphorus homeostasis, elevates oxidative stress, and disrupts the antioxidant defense system. Histological and SEM analyses reveal extensive damage to the trabecular bone architecture, reinforcing the harmful effects of the venom on skeletal health. These results underscore the need for further research to better understand the acute and long-term implications of *B. atrox* envenoming, particularly regarding its potential impact on human bone.

## Highlights

• Research into *Bothrops atrox* venom effect on bone remodeling, and calcium and phosphate homeostasis remain limited.
• *B. atrox* venom induced significantly increase in plasma calcium levels.
• *B. atrox* venom significantly reduced calcium and phosphorus levels in bone mice.
• *B. atrox* venom induces stress homeostasis disorders in bone tissues in mice.
• *B.* *atrox* envenoming caused bone rarefaction confirmed by histology and scanning electron microscopy analysis.

## Graphical abstract

**Figure:**
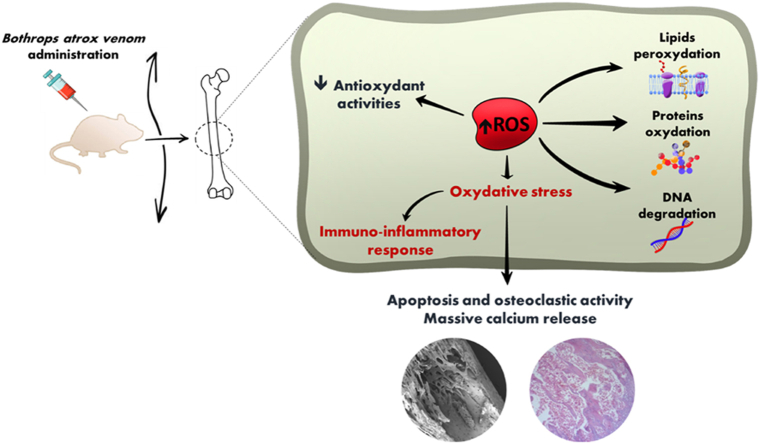

## 1 Introduction

*Bothrops atrox* is a pit viper species widely prevalent across South America (Monteiro et al., 2020). Its venom can trigger a wide array of local and systemic manifestations (Ibiapina et al., 2019; Monteiro et al., 2020; S Oliveira et al., 2020; Silva et al., 2021). These effects result from both the direct action of the venom, the ensuing immune and inflammatory responses, and the distortion of redox homeostasis in snake victims (Gutiérrez et al., 2017; Resiere et al., 2022). The reported toxic effects of *B. atrox* venom include coagulation disorders, myotoxicity, nephrotoxicity, and localized tissue degradation (de Souza et al., 2012; Santhosh et al., 2013; Sunitha et al., 2015).

*B. atrox* venom contains a wide array of toxin components, including metalloproteinases (SVMPs), serine proteases (SVSPs), phospholipases A_2_ (PLA_2_s), hyaluronidases (Hyals), L-amino acid oxidases (LAAOs), and many others. SVMPs are classified into three structural subclasses (PI, PII, and PIII) based on their domain composition (Lopes-de-Souza et al., 2023). In *B. atrox* venom, the main isolated subclasses are PI and PIII (Larréché et al., 2023). They can disrupt the extracellular matrix and basement membrane by degrading collagen and other structural proteins (Gutiérrez and Rucavado, 2000). PLA_2_s contribute to cytotoxicity by disrupting membrane integrity and triggering an inflammatory response (Hiu and Yap, 2020). The LAAOs from *B. atrox* venom can trigger cellular apoptosis through hydrogen peroxide (H_2_O_2_) generation during enzymatic activity (Costal-Oliveira et al., 2019). Synergistic interactions among these major toxins further potentiate the venom's overall pathogenic effects.

Calcium is one of the most abundant minerals in the body, with over 99 % stored in bone as hydroxyapatite crystals. This mineralized form provides structural strength to the skeleton and serves as a dynamic reservoir for maintaining serum calcium homeostasis during the lifespan. Bone itself is a composite tissue comprising cellular elements (osteoblasts, osteocytes, osteoclasts, and bone lining cells), mineral components (calcium and phosphate), and an organic matrix of collagen and non-collagen proteins (Mohamed, 2008). Bone remodeling is a continuous and dynamic process through which bone tissue is renewed to maintain skeletal integrity and mineral balance. It relies on a coordinated activity between osteoclasts (bone resorption and calcium and phosphate release into the bloodstream) and osteoblasts (new bone matrix synthesis). An imbalance between osteoclast and osteoblast activity can impair the phosphate and calcium homeostasis and bone structure. Overall, calcium hemostasis is ensured by complex interactions involving the gut, kidneys, and bone and is regulated by signaling pathways involving hormones like parathyroid hormone (PTH), calcitonin, growth factors, sex hormones, and others (Hadjidakis and Androulakis, 2006).

In snakebite envenoming victims, extracellular Ca^2+^ plays a key role in mediating the toxic effects. It is a critical determinant of the regulation of the clotting pathway (James and Roche, 2004; Mann, 2017). Furthermore, a study on *Bothrops asper* venom revealed that calcium protects the plasma membrane from the cytotoxic action of myotoxin II, a Lys49 phospholipase A_2_ homologues from this venom (Villalobos et al., 2007). However, the plasma membrane disruption by the studied toxins in this study can induce a prominent Ca^2+^ influx into the muscular cells, responsible for cellular apoptosis and death (Mora et al., 2006). While numerous studies have explored the toxicological effects of *B. atrox* venom on various organs and systems (Cavalcante et al., 2021; Gutiérrez et al., 2017; Ibiapina et al., 2019; Kallel et al., 2024; Monteiro et al., 2020; S Oliveira et al., 2020; Silva et al., 2021), there remains a notable gap in research regarding its impact on calcium homeostasis and bone remodeling. This study aimed to investigate the acute effects of *B. atrox* venom on calcium homeostasis and bone tissue in mice.

## 2 Material and methods

### 2.1 Venom and mice

Freeze-dried venom from *B. atrox* was sourced from Latoxan (Portes-lès-Valence, France). Venom samples correspond to pools obtained from several male and female adult specimens, from French Guiana.

Both male and female Adult Swiss mice were obtained from the Tunisian Central Pharmacy (SIPHAT, ISIN: TN0006670012, Ben Arous B 14743 1996, Tunisia), weighing approximately 30 ± 2 g/mouse. Mice were housed in an air-conditioned room maintained at 22 ± 3 °C with a relative humidity of 40 %. They were kept in polycarbonate cages (40 × 25 × 20 cm) and had unrestricted access to a standard pellet diet (“SNA” Animal Nutrition Company, Sfax, Tunisia) and water.

The experimental procedures adhered to the European Directive 2010/63/EU of September 22, 2010, concerning the protection of animals used for scientific purposes. All protocols were reviewed and approved by the Ethical Committee of the Higher Institute of Biotechnology, University of Sfax, Tunisia (Protocol No. 09.0010/22).

### 2.2 Experimental design

We investigated two groups of 6 female and two groups of 6 male mice. The control groups (6 male and 6 female mice) received phosphate-buffered saline solution (PBS) (pH = 7.2), while the treated groups (6 male and 6 female mice) received intraperitoneal (IP) injection 1/2 of the LD_50_ of *B. atrox* venom corresponding to 25.4 μg/male mouse and 31.3 μg/female mouse (Kallel et al., 2024). After 24 h of venom or PBS injection (the acute phase), mice were sacrificed by cervical dislocation.

### 2.3 Blood and urine sampling

Blood samples were collected in heparinized tubes and centrifuged at 2200×*g* for 15 min to obtain plasma. Urine samples were collected using metabolic cages. Both plasma and urine were stored at −80 °C for subsequent mineral analysis.

### 2.4 Bone samples' preparation

Femurs were dissected out, and the surrounding muscles and connective tissues were removed.

For laboratory tests, femurs were rinsed, pulverized into powder, homogenized (100 mg/mL) at 4 °C in 0.1 mol/L Tris–HCl buffer (pH 7.4), and centrifuged at 3000 g for 10 min. Bone homogenate was stored at −80 °C for subsequent mineral composition and oxidative stress analyses.

For histopathology, femurs from male and female mice were immediately fixed in BB's (ethanol 95°, 40 % of formaldehyde, and water) for 48 h. Then, they were decalcified with 10 % nitric acid, dehydrated, and embedded in paraffin blocks. Finally, these blocks were sectioned at 4 μm thick and stained with hematoxylin and eosin (H-E).

### 2.5 Assessment of calcium and phosphorus balance

Calcium and phosphorus levels in plasma, urine, and bone (after nitroperchloric mineralization) were assayed by colorimetric techniques (Cobas 6000, Roche®) and expressed as mg/L.

### 2.6 Oxidative stress measurement in bone

#### 2.6.1 ROS measurement

ROS levels in the bone homogenate were measured using 20,70-dichlorofluorescein diacetate (DCFH-A) with ice-cold Locke's buffer (154 mM NaCl, 5.6 mM KCl, 3.6 mM NaHCO_3_, 2.0 mM CaCl_2_, 10 mM D–glucose and 5 mM [4–(2–hydroxyethyl) 1–piperazine] ethane sulfonic acid (HEPES), pH 7.4). DCFH-DA (5 mM) was added to the bone homogenate and incubated for 30 min at 37 °C in the dark. The fluorescence intensity was measured using a fluorescence plate reader with an excitation wavelength of 485 nm and emission detection at 530 nm (Feki et al., 2023). ROS dosage was expressed in μmol/mg protein.

#### 2.6.2 Determination of malondialdehyde level

Lipid peroxidation (LPO) in the bone homogenate was estimated by measuring thiobarbituric acid–reactive substances (TBARS). It was expressed in terms of Malondialdehyde (MDA) content, which is the end product of LPO (Buege and Aust, 1978; Jebahi et al., 2013). Technically, a bone homogenate sample (1.0 ml) was mixed with 2.0 ml of a solution of 15 % w/v of trichloroacetic acid (TCA) and 0.375 % w/v of TBA prepared in 0.25 N HCI. The mixture was heated for 15 min in a boiling-water bath and then cooled and centrifuged at 1500×*g* to remove the precipitate of the tissue sample. The MDA concentration of the supernatant was determined directly from the molar extinction coefficient of the pink pigment at 535 nm absorption and was expressed in nmol/g protein.

#### 2.6.3 Antioxidant enzymes and non-enzymes assessment

The superoxide dismutase (SOD) activity in the bone homogenate was assayed by the spectro-photometric method (Marklund and Marklund, 1974). Reduced glutathione (GSH) was measured at 412 nm using the method described by Jollow et al. (1974). The process is based on the development of a yellow color when 5,5-dithiobtis-2 nitro benzoic acid (DTNB) is added to the compounds containing sulphydryl groups. SOD was expressed as U/mg protein. Total GSH content was expressed as mg/g of protein. Glutathione peroxidase (GPx) activity was determined as described by Flohé and Günzler (1984). The absorbance at 340 nm was recorded, and the GPx enzyme activity was expressed as nmoles of GSH oxidized/min/mg protein. The level of total proteins was determined according to Lowry et al. (1951).

### 2.7 Scanning electron microscopy

Scanning electron microscopy (SEM) was performed on the operated femurs of control and treated mice. Briefly, the femurs were longitudinally cut and immersed in 50 % sodium hypochlorite to discard organic material. After 3 h, bone samples were rinsed for 30 min in distilled water and fixed overnight in 1 % osmium tetroxide dissolved in 0.1 M cacodylate buffer (pH 7.2). Then, samples were rinsed for 30 min in distilled water and dehydrated in ascending series (70 %, 95 %, and 100 %) of ethanol. Finally, samples were conducted for 1 h in hexamethyl disilazane, followed by air drying using a filter paper. They were analyzed by SEM at 20 kV (SEM JEOL JSM-5100).

### 2.8 Histomorphometric analysis of bone samples

The following parameters were measured in bone samples: trabecular bone volume (BV/TV, in %), trabecular thickness (Tb.Th, in μm), trabecular number (Tb.N, in mm^−1^), intertrabecular distance (Tb.Sp, in μm), and osteoid surface (OS/BS, in %). The latter determination was mainly based on the percentage of endosteal bone surface, presenting features of bone resorption with osteoclasts.

### 2.9 Statistical analysis

Quantitative variables were expressed as mean ± SD and compared using Mann et Whitney test. Differences were considered significant when p < 0.05. Statistical analyses were carried out using Excel (2010 Microsoft Corporation, Redmond, USA) and IBM SPSS Statistics for Windows, version 24 (IBM Corp., Armonk, N.Y., USA). Bone histomorphometry of mice femurs was assessed using Image J. 1.48v software (Strissel et al., 2007).

## 3 Results

### 3.1 Calcium and phosphorus balance

In controls, calcium dosage in plasma was 62.7 ± 4.3 mg/L in male and 62 ± 0.9 mg/L in female mice (p = 0.818). Phosphorus dosage in plasma was 79.5 ± 9.9 mg/L in male and 73.7 ± 5.9 mg/L in female mice (p = 0.310). Calcium dosage in bone was 73.3 ± 7.6 mg/L in male and 70.2 ± 3.2 mg/L in female mice (p = 0.818). Calcium dosage in urine was 77.3 ± 3.6 mg/L in male and 74.7 ± 3.6 mg/L in female mice (p = 0.240).

In male mice injected with *B. atrox* venom, mean plasma calcium levels significantly increased while phosphorus levels decreased compared to controls (+54.2 % and −52.4 %, respectively, p = 0.002 for both). Similarly, in female mice, mean plasma calcium levels significantly increased while phosphorus levels decreased compared to controls (+29 % and −39.3 %, respectively, p = 0.002 for both). Regarding bone analysis, *B. atrox* venom injection reduced calcium levels (−66.8 % and −41.6 %, in male and female mice, respectively, compared to controls, p = 0.002 for both). Concomitantly, *B. atrox* venom injection resulted in a significant decrease in urine calcium excretion (−63.1 % and −51.4 %, in male and female mice, respectively, compared to controls, p = 0.002 for both) and a significant increase in urine phosphorus excretion (+46 % and +44.6 %, in male and female mice, respectively, compared to controls, p = 0.002 for both).

Overall, variations in calcium and phosphorus levels in plasma and bone were significantly more pronounced in male than in female mice (Table 1).

**Table 1:** Calcium, phosphorus, and oxidative stress parameters in control and *B. atrox* envenomed mice.

	Control mice			Envenomed mice				
	Male	Female	p_1_	Male	Female	p_2_	p_3_	p_4_
Ca^2+^ in plasma (mg/L)	62.7 ± 4.3	62 ± 0.9	0.818	96.7 ± 4.7	80 ± 1.9	0.002	0.002	0.002
Ph in plasma (mg/L)	79.5 ± 9.9	73.7 ± 5.9	0.310	37.8 ± 3.7	44.7 ± 5.6	0.041	0.002	0.002
Ca^2+^ in bone (mg/L)	73.3 ± 7.6	70.2 ± 3.2	0.818	24.3 ± 4.1	41 ± 3.7	0.002	0.002	0.002
Ph in bone (mg/L)	107.1 ± 16.3	95.2 ± 5.3	0.394	32.5 ± 3.7	41.8 ± 3.5	0.002	0.002	0.002
Ca^2+^ in urine (mg/L)	77.3 ± 3.6	74.7 ± 3.6	0.240	28.5 ± 9.4	36.3 ± 4.1	0.132	0.002	0.002
Ph in urine (mg/L)	169.7 ± 14	162.5 ± 10.8	0.394	247.7 ± 6.9	235 ± 21.7	0.310	0.002	0.002
ROS (μmol/mg protein)	100 ± 0	100 ± 0	1.000	229.2 ± 15.6	200 ± 14.1	0.009	0.002	0.002
MDA (nmol/g protein)	23.1 ± 1.3	24.7 ± 1.8	0.132	64.9 ± 3.4	62 ± 2.7	0.180	0.002	0.002
SOD (U/mg protein)	19 ± 0.4	18.8 ± 0.6	0.589	9.4 ± 1.1	31.7 ± 1	0.002	0.002	0.002
GPx (nmol of GSH/mg protein)	12 ± 0.6	11.9 ± 0.5	0.589	2.6 ± 0.5	5.1 ± 0.3	0.002	0.002	0.002
GSH (mg/g protein)	62.4 ± 1.8	64.7 ± 1.5	0.026	21.9 ± 1.7	27.8 ± 1.9	0.002	0.002	0.002

p_1_: Male *vs.* Female in the control group; p_2_: Male *vs.* Female within the envenomed mice; p_3_: Envenomed *vs.* Control mice in the male group; p_4_: Envenomed *vs.* Control mice in the female group.

### 3.2 Effects of venom on bone oxidative stress parameters

#### 3.2.1 ROS and MDA levels

In controls, ROS dosage in bone homogenate was 100 ± 0 μmol/mg protein in male and female mice (p = 1.000). MDA dosage in bone homogenate was 23.1 ± 1.3 nmol/g protein in male and 24.7 ± 1.8 nmol/g protein in female mice (p = 0.132). After *B. atrox* venom injection, ROS levels in bone tissues increased significantly in both male and female mice (+129 % and +100 %, respectively, p = 0.002 for both), compared to control groups. Notably, this elevation was markedly more pronounced in male than female mice (p = 0.009). Furthermore, MDA levels in bone tissues also demonstrated significant increases in both sexes (+181 % and +151 % in male and female mice, respectively, p = 0.002 for both), relative to controls (Table 1). Importantly, no significant difference was noted between the male and female groups regarding MDA variation (p = 0.180).

#### 3.2.2 Enzymatic and non-enzymatic antioxidant status

In controls, SOD dosage in bone homogenate was 19 ± 0.4 U/mg protein in male and 18.8 ± 0.6 U/mg protein in female mice (p = 0.589). GPX dosage in bone homogenate was 12 ± 0.6 nmol of GSH/mg protein in male and 11.9 ± 0.5 nmol of GSH/mg protein in female mice (p = 0.589). GSH dosage in bone homogenate was 62.4 ± 1.8 mg/g protein in male and 64.7 ± 1.5 mg/g protein in female mice (p = 0.026). *B. atrox* venom injection resulted in a significant decrease in SOD activities in male mice (−50.5 %; p = 0.002), while SOD activities were increased in female mice (+68.6 %; p = 0.002), when compared to controls (Table 1). Concurrently, GPx and GSH activities decreased in male (−78.3 % and −64.9 %, respectively, p = 0.002 for both) and female mice (−57.1 % and −57 %, respectively, p = 0.002 for both) (Table 1). Notably, the decreases in GPx and GSH activities were more pronounced in male than in female mice (p = 0.002 for both).

### 3.3 Histological findings

**Fig. 1:**
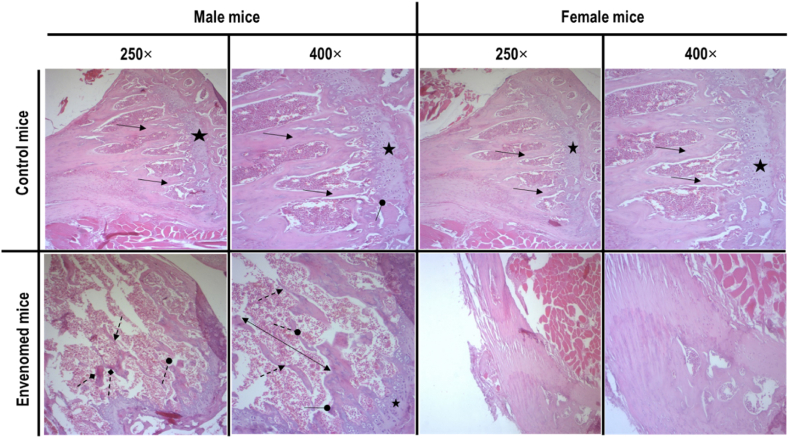
Hematoxylin and eosin (H–E) stained sections of epiphysis with a normal appearance from control and treated (*B. atrox* venom) male and female mice. All sections showed severe bone rarefaction. Original magnification of H-E stain (250x and 400x).Symbols indicate:: Continuous trabecular bone, : Discontinuity of trabecular bone, : Disappearance of nodes, : Compact bone with normal appearance, : Increased intertrabecular spaces, : Bone necrosis.

Histological examination of the epiphyses in male and female control groups showed a standard and mature bone matrix with normal hematopoietic tissue and a normal organization of the epiphyseal cartilage (Fig. 1). However, in mice receiving *B. atrox* venom, we observed bone rarefaction, confirmed by the discontinuities of the trabecular spans and disappearance of nodes in male and female mice as compared to controls (Fig. 1).

### 3.4 Scanning electron microscopy analysis

**Fig. 2:**
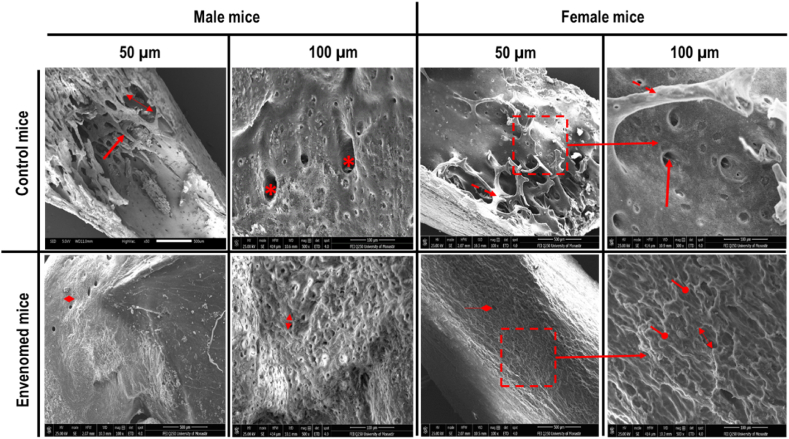
SEM micrographs of femurs from control and treated (*B. atrox* venom) male and female mice showing a homogeneous and normal aspect of the bone in controls and rarefaction of trabecular bone with decreased area osteon of compact bone in mice exposed to *B. atrox* venom. Symbols indicate:: Normal osteon of compact bone : Normal trabecular traverse in bone trabecular : Thinning and discontinuity of trabecular traverse : Decrease of area osteon of compact bone : Intertrabecular distance.

SEM images of the femur from control male and female mice show a homogeneous and regular aspect of the trabecular and compact bone (Fig. 2). However, in mice exposed to *B. atrox* venom, we observed rarefaction of trabecular bone with decreased area osteon of compact bone (Fig. 2).

### 3.5 Bone histomorphometry

Data obtained from bone histomorphometry showed significant changes in male and female bones exposed to *B. atrox* venom compared to control mice (Table 2). Administration of *B. atrox* venom caused a substantial decrease in osteoid surfaces (OS/BS: 28.3 % and −39.4 %, p = 0.008 for both) without significant changes in trabecular thickness (Tb.Th: 16.8 % and −16.1 %, p = 0.095 and p = 0.056), trabecular bone volume (BV/TV: 1.7 % and −7.5 %, p = 1 and p = 0.690), and trabecular number (Tb.N: +20.7 % and −16.1 %, p = 0.222 for both), and intertrabecular distance in trabecular bone (Tb.Sp: 25.2 % and −25.1 %, p = 0.095 and p = 0.151) in male and female mice, respectively.

**Table 2:** Bone histomorphometry in control and *B. atrox* envenomed mice.

	Control mice			Envenomed mice				
	Male	Female	p_1_	Male	Female	p_2_	p_3_	p_4_
Trabecular bone volume (%)	65.8 ± 14.1	62.5 ± 15.6	0.421	64.7 ± 13.9	57.8 ± 5.5	0.548	1.000	0.690
Trabecular thickness (μm)	56 ± 5.5	54.5 ± 8.4	0.310	46.6 ± 6.5	45.7 ± 3.9	1.000	0.095	0.056
Trabecular number (mm^−1^)	8.2 ± 2	9.3 ± 4.2	0.548	9.9 ± 1.6	7.8 ± 0.6	0.008	0.222	0.222
Intertrabecular distance (μm)	50.6 ± 12.4	45.4 ± 13.2	0.841	37.8 ± 6.8	34 ± 2.6	0.222	0.095	0.151
Osteoid surface (%)	9.2 ± 0.9	9.9 ± 1.5	0.421	6.6 ± 0.3	6 ± 0.6	0.056	0.008	0.008

p_1_: Male *vs.* Female in the control group; p_2_: Male *vs.* Female within the envenomed mice; p_3_: Envenomed *vs.* Control mice in the male group; p_4_: Envenomed *vs.* Control mice in the female group.

## 4 Discussion

The acute effects of *B. atrox* venom on bone tissue were investigated in male and female mice following intraperitoneal injection. Calcium homeostasis was assessed through plasma, urine, and bone calcium and phosphorus levels, along with evaluations of redox status and histological alterations in bone. *B. atrox* envenoming induced marked disruptions in phosphocalcic balance and redox homeostasis, accompanied by severe bone abnormalities as revealed by histology, scanning electron microscopy, and bone histomorphometry. These metabolic and histological changes were more pronounced in males than in females. To our knowledge, this is the first study examining the acute impact of *B. atrox* venom on calcium homeostasis and bone tissue in mice.

Following *B. atrox* venom injection (24 h), we observed a significant increase in plasma calcium levels and decreased phosphorus plasma levels in mice compared to controls. Notably, phosphocalcic balance changes were more pronounced in male than female mice. These changes were concomitant with a decrease in bone mineral density and overall bone quality. In cases of *Bothrops* envenoming, hypercalcemia can be explained by an intense bone cell apoptosis and death, resulting in an intense release of calcium content into the bloodstream. Hypophosphatemia typically accompanies hypercalcemia (Matikainen et al., 2021). In addition, our study documented severe structural bone damage, which can be explained by massive bone cells apoptosis, and may underlie the intense calcium release and subsequent hypercalcemia.

Physiologically, the modulation of urinary calcium excretion is essential for maintaining calcium homeostasis. Typically, hypercalcemia is associated with increased urinary calcium excretion mediated by the PTH (Matikainen et al., 2021). The latter facilitates calcium mobilization from bone and stimulates calcium reabsorption at the tubular level, while it decreases phosphate reabsorption (Khan et al., 2025). In case of hypercalcemia, PTH in circulation would be suppressed (negative feedback), leading to decreased Ca^2+^ and increased phosphate reabsorption at the renal tubular level. In our study, we observed an increase in Ca^2+^ and a decrease in phosphate levels in plasma, coupled with a dramatic decrease in both in bone. This suggests a direct effect of the venom on bone tissue, resulting in significant calcium release and a negative feedback mechanism that suppresses PTH secretion. Overall, the concomitant paradoxical hypocalciuria and hyperphosphaturia raise questions about the involvement of some hormonal pathways and the venom-induced tubular toxicity.

Oxidative stress is a key process driving cell necrosis after *B. atrox* envenoming, and the strong production of H_2_O_2_ via LAAO catalytic reaction may be a contributing factor (Costal-Oliveira et al., 2019). Redox disturbances can affect bone remodeling, causing an imbalance between osteoclast and osteoblast activity (Domazetovic et al., 2017). This may lead to alterations in bone mineral density, structural integrity, and phosphocalcic metabolic regulation. In our study, *B. atrox* venom exposure produced oxidative stress in mice bones, evidenced by increased ROS and MDA production, and significant depletion of antioxidant defenses (GPx and GSH), likely consumed in neutralizing oxidant radicals. Interestingly, SOD decreased in males, but increased in female mice demonstrating that females continue to defend against excess ROS, while the antioxidant reserves of males are totally depleted. These oxidative changes may contribute to cell apoptosis, bone tissue damage, and calcium homeostasis disorders.

The histological examination and SEM findings on bone in mice exposed to *B. atrox* venom revealed an outstanding bone rarefaction with discontinuities of trabecular bone, disappearance of nodes, and bone necrosis. Considering the femoral histomorphometry parameter changes, the osteoid surface was decreased in mice injected with *B. atrox* venom. These findings reflect a disruption in bone remodeling resulting in bone rarefaction and reduced wall thickness in trabecular bone. Based on the biological and histopathological findings, we hypothesize a complex mechanism associating: (1) a direct toxicity of the venom on bone cells mediated by the venom enzymes, like SVMPs and PLA_2_; (2) an inflammatory reaction mediated by PLA_2_ especially that bone structure is rich in inflammatory cells (Mohamed, 2008); and (3) redox disturbances with lipid peroxidation and protein carboxylation reactions.

Overall, the principal *B. atrox* venom components implicated in bone damage are SVMPs, which degrade extracellular matrix proteins, PLA_2_s, which hydrolyze phospholipids in cell membranes and the extracellular matrix, releasing arachidonic acid and triggering inflammatory responses, and LAAO which contribute to redox disturbances (Costal-Oliveira et al., 2019; Gutiérrez and Rucavado, 2000). Collectively, the activity of these enzymes suggests a direct effect on the bone matrix and a substantial ROS assault, potentially compromising osteocyte integrity and further influence bone remodeling. Moreover, *Bothrops moojeni* and Cobra venoms can inhibit osteoclast differentiation, bone resorption, and cytoskeletal organization (D’Amélio et al., 2025; Wu et al., 2006). Therefore, the effects of *B. atrox* venom on bone could be multifactorial involving direct matrix proteolysis, osteocytes apoptosis, and cellular modulation of osteoclast function.

In a previous study, we demonstrated that male mice exhibit greater susceptibility to *B. atrox* venom than females. (Kallel et al., 2024). We suggested that sex-related differences in toxic manifestations induced by venom in mice must be considered when conducting experimental studies. In the current study, calcium and phosphorus changes in plasma and bone homogenate were significantly more pronounced in male than in female mice. Similarly, ROS, enzymatic and non-enzymatic antioxidants changes were more significant in males than females. These results converge toward the higher susceptibility of male than female mice to *B. atrox* venom, probably in relation to genetic and hormonal parameters (Cai et al., 2016; Kallel et al., 2024; Klein and Flanagan, 2016).

We acknowledge several limitations in our study. First, while the exact composition of the *B. atrox* venom batch used was not fully characterized, its overall enzymatic profile is well-established, with only potential variations in the relative abundance of individual components (Sousa et al., 2013, 2021). Second, we measured total serum calcium levels without assessing ionized calcium, which may provide a more accurate representation of calcium status. Third, we used ½ LD_50_ venom doses rather than gradually increased ones to investigate the initial venom impact on bone and phosphocalcic homeostasis. Fourth, our study focused on the acute effects of *B. atrox* venom on bone without examining the long-term consequences of smaller venom doses. Fifth, extrapolating these findings to human bone tissue is questionable due to the discrepancy between experimental LD_50_ and real-world snakebite venom exposure. Finally, studying calcium homeostasis and the bone impact of snake venom requires a deeper investigation of kidney, hormonal, and hematopoietic parameters to ensure a holistic approach to the disease. Nonetheless, this represents the first investigation into the impact of *B. atrox* venom on bone tissue and phosphocalcic equilibrium.

## 5 Conclusion

This study provides novel insights into the toxicological effects of *Bothrops atrox* venom on bone tissue in mice. The findings highlight significant disruptions in mineral homeostasis, including hypercalcemia and decreased phosphorus levels. These disturbances were particularly pronounced in male mice, suggesting a sex-dependent susceptibility to the venom. Additionally, the observed redox disturbances underscore the role of oxidative damage in venom-induced bone toxicity. Histological and SEM analyses further confirmed the adverse effects on bone structure, including bone rarefaction, trabecular discontinuities, and necrosis.

Finally, this study provides the first detailed exploration of *B. atrox* venom's impact on bone tissue, revealing its multifaceted toxicity and significant implications for bone health and systemic mineral metabolism. These findings underscore the need for further research to better understand the acute and long-term consequences of *B. atrox* envenomation, particularly regarding its potential impact on human bone health.

## CRediT authorship contribution statement

**Hatem Kallel:** Writing – review & editing, Supervision, Project administration, Methodology, Investigation, Conceptualization. **Latifa Hamdaoui:** Methodology, Investigation. **Malek Elerou:** Writing – original draft, Methodology, Investigation. **Marwa Lakhrem:** Writing – original draft, Methodology, Investigation. **Stephanie Houcke:** Writing – review & editing, Methodology. **Majed Kammoun:** Writing – original draft, Methodology. **Dabor Resiere:** Writing – review & editing, Methodology. **Tarek Rebai:** Writing – original draft, Investigation, Conceptualization. **Jean Marc Pujo:** Writing – review & editing, Investigation, Conceptualization. **Ibtissem Ben Amara:** Writing – review & editing, Writing – original draft, Validation, Supervision, Project administration, Methodology, Investigation, Conceptualization.

## Ethical statement

The authors certify that.

- This manuscript was approved by all authors before submission.
- The study was carried out according to the “Directive 2010/63/EU of the European Parliament and of the Council of September 22, 2010 on the protection of animals used for scientific purposes” and was approved by the Higher Institute of Biotechnology (University of Sfax, Tunisia) Ethical Committee (Protocol n° 09.0010/22).
- The article does not contain any personal data of human subjects.

## Declaration of competing interest

The authors declare that they have no known competing financial interests or personal relationships that could have appeared to influence the work reported in this paper.

## Data Availability

Data will be made available on request.
